# COVID-19 quarantine: Post-traumatic stress symptomatology among Lebanese citizens

**DOI:** 10.1177/0020764020932207

**Published:** 2020-06-03

**Authors:** Mirna Fawaz, Ali Samaha

**Affiliations:** 1 Nursing Department, Faculty of Health Sciences, Beirut Arab University, Beirut, Lebanon; 2 Faculty of Public Health IV, Lebanese University, Zahle, Lebanon; 3 Faculty of Letters and Human Sciences IV, Lebanese University, Zahle, Lebanon; 4 Department of Biomedical Sciences, Lebanese International University, Beirut, Lebanon

**Keywords:** Coronavirus, PTSS, COVID-19, pandemic, quarantine

## Abstract

**Background:**

In the light of the global spread of the novel Coronavirus known as COVID-19 and in the absence of an approved treatment and vaccination, Lebanon has taken national measures, among which was home quarantine of the general public in an attempt to flatten the epidemic curve and avoid flooding the health care system.

**Aim:**

This study aimed at evaluating the prevalence of post-traumatic stress symptomatology (PTSS) during the times of COVID-19 quarantine among Lebanese citizens.

**Method:**

This quantitative cross-sectional study recruited 950 civilians and is aimed at measuring the prevalence of PTSS among the Lebanese citizens at an interval of 2 weeks and 1 month of COVID-19 quarantine.

**Results:**

The results have shown that quarantine in Lebanon has started to give rise to Post-traumatic Stress Disorder symptomatology during the second week which was worsened in the fourth week of COVID-19 quarantine.

**Conclusion:**

COVID-19 quarantine has influenced the psychology of Lebanese citizens and might have persistent effects after the end of this phase which is recommended to be explored.

## Introduction

Quarantine is the segregation and limitation of travel of individuals who might have been subjected to an infectious illness to decide if they are ill, thus decreasing their chance of contaminating others (Centers for Disease Control and Prevention, 2020). This concept varies from isolation, which is the seclusion of those who have been afflicted with an infectious illness from healthy uncontaminated individuals; however, the two definitions are sometimes used synonymously, particularly in interaction with the media. The term quarantine was first recorded in 1127 in Venice, Italy, and became commonly employed in reaction to the Black Death, but it was not until 30 decades later that the United Kingdom formally began to enforce a quarantine in answer to the epidemic (Newman, 2012).

Quarantine has been used in response to the Coronavirus disease 2019 (COVID-19) outbreak. To date, the world has recorded 382,126 Coronavirus cases, with 16,568 deaths, and 102,501 recovered cases. This epidemic witnessed whole cities in China immediately put under national quarantine, while hundreds and thousands of foreign citizens arriving in China were told to protect themselves at households or in government facilities (Zhao & Chen, 2020). Most recently, upon declaring the COVID-19 as a worldwide pandemic by the World Health Organization (WHO), many countries around the world besides China, have been put under quarantine and have been enforcing social distancing measures through health policy authorities in an attempt to ‘flatten the curve’, reduce the outbreak and flooding the health care system as a consequence (Wu & McGoogan, 2020).

These initiatives have precedence, where during the 2003 epidemic of a severe acute respiratory syndrome (SARS), statewide quarantines have also been placed in regions of China and Canada, while whole communities in several West African nations were quarantined during most of the 2014 Ebola epidemic (McCoy, 2016). To date, Lebanon has recorded 717 cases of COVID-19 with 24 deaths, where amid economic crisis (WHO, 2020), the Ministry of Public Health in Lebanon has declared the state of emergency approximately 2 weeks after the appearance of the first case of COVID-19, after the national press has launched nationwide campaigns which declared a state of civil emergency, which have incentivized the populace to stay homebound. The country has closed all public and private sector institutions including educational, food service, and leisure institutions, thus placing the whole state under lockdown and the people in home quarantine, where the armed forces have taken on the responsibility of maintaining (Hopman et al., 2020).

Quarantine, nevertheless, can be an inconvenient activity for those who undertake it. Segregation from family members, lack of rights, confusion about the state of the illness, and fatigue may trigger drastic results many times. Suicide has been documented, major anger has been created and litigation brought after the quarantine was enforced in previous epidemics (Barbisch et al., 2015). The viability and efficacy of COVID-19 propagation in domestic and community environments have been studied (WHO, 2020) and yet the perception of those put under quarantine in terms of adherence, challenges, emotional reaction, and mental consequences remains under-researched (Wilder-Smith et al., 2020).

The possible advantages of compulsory collective quarantine also ought to be closely measured against the potential psychological risks (Rubin & Wessely, 2020). Effective usage of quarantine as a method of public safety demands one to observe and reduce the adverse consequences correlated with it to the degree necessary. Therefore, considering the emerging situation with coronavirus, policymakers desperately need a convergence of evidence to provide recommendations for the public. In situations like this, WHO (2020) suggests fast evaluations.

Certainly symptomatic persons would be undergoing stress because of their concerns about COVID-19 developing, the possibility of dying, and the potential for exposing others. This stress is expected to be intensified due to the prolonged duration of isolation or quarantine (Brooks et al., 2020). This study aims at investigating the psychological effect of the quarantine and mainly the prevalence of post-traumatic stress symptomatology (PTSS) during the times of COVID – among Lebanese citizens. Post- traumatic stress symptomatology (PTSS) accompany stressful events beyond the context of normal human encounters, such as aggressive sexual attacks, abuse, injuries, violence or natural catastrophes, and are defined by standard intrusive manifestations, the intensity of distress, subsequent stimuli avoidance, affective numbing and hyperarousal of certain physiological functions (Deja et al., 2006). Post-traumatic stress (PTS) defines a broad collection of symptoms that an individual may experience after living through an incredibly stressful event. However, Post-traumatic Stress Disorder (PTSD) is an official diagnosis with more intense and continued symptoms being prevalent. Such a diagnosis would take at least 6 months to make. Identifying the prevalence of PTSS is quite significant to prevent further development and complication of symptoms into disorders. In addition, such research would provide the scientific community with ethnically relevant data regarding the psychological response to quarantine among asymptomatic Lebanese citizens, whether they might have been exposed or have been practicing social distancing to avoid exposure. These data would give the health authorities more insight regarding the proper measures to be taken in order to make such national enforcements successful and sustainable, thus affecting health communication and public awareness campaigns and control measures used by the government in a way which will, in turn, increase compliance among citizens and enhance their emotional and psychological coping with the situation.

## Methods

The study employed a quantitative cross-sectional research design. An online questionnaire was sent through a google form link via email to be completed by quarantined people in different Lebanese geographic areas. The questionnaire was sent to 1,067 citizens, where their emails were accessed through university web-mails and national syndicates, where we received an 89.03% response rate. The analysis was conducted based on a sample of 950 respondents belonging to various demographic profiles and from various provinces of Lebanon. The participants included in this study were adult community living civilians who have abided by home quarantine and social distancing which is characterized by staying home and not leaving unless if there is a need, keeping a distance of 1–3 meters between oneself and anyone who is coughing or sneezing (WHO, 2020). Quarantine enforced in Lebanon was characterized by only going out to the supermarket or the pharmacy, no retail shops, no restaurants or cafeterias, no availability of public transportation, using private vehicles was only allowed on certain fixed dates stated by the government between the hours of 5 am and 7 pm only which were the imposed curfew hours. Citizens were not able to go out during the light of day for unnecessary purposes which are usually monitored by law enforcement officials; however, visiting families was doable from time to time. It was the situation of a partial embargo enforced by the government and military forces. The study excluded any person who is exhibiting any signs of coronavirus infection or who has been diagnosed to have COVID-19 or any person who is diagnosed with any mental illness, in order to isolate and study the effect of home quarantine on the psychological status of the citizens. Institutional Review Board approval was procured as the study has abided by the ethical guidelines of research (IRB number: ECO-R-12). The online questionnaire was sent to the eligible participants on 18 March 2020 and then on 1 April 2020, and written informed consent was requested in correspondence. The participants received an explanation of the study’s aim and that all the data extracted will be confidential noting that the participation is voluntary. The data were collected from the same participant on two occasions; 2 weeks and then 4 weeks after the start of quarantine. The survey included a sociodemographic data sheet which also measured the participant’s certain behavior pattern of quarantine. The second part of the survey included the PTSD Checklist–Civilian Version (PCL-C) which measured the psychological experiences during the quarantine period. PTSD symptom severity was assessed using the 17-item PCL-C (Weathers et al., 1993). The PCL-C is made of up B, C and D items, where B items relate to the active symptoms of PTSD, C items pertain to what is called numbing symptoms and are characterized by avoidance and passivity, and the D items which are referred to as hyperarousal or hyperactive symptoms (Simms et al., 2002). The PCL has been used in diverse samples including hospitalized physical injury survivors and possesses solid psychometric properties (Ruggiero et al., 2003; Wang et al., 2010). Participants rated the degree to which they were bothered by each symptom on a scale ranging from 1 (*not at all*) to 5 (*extremely*), with possible scores ranging from 17 to 85. The reliability of the PCL-C was examined by the research where a Cronbach’s alpha of .88 was recorded. The survey’s reliability and use were also validated by previous research where a Cronbach’s alpha of .90 was recorded by Gelaye et al. (2017). Upon completion of the survey, the data were entered into SPSS version 22 for analysis where descriptive and inferential statistics were carried out.

## Results

### Sociodemographic data and quarantine practices

The sample of this study comprised of 292 (30.7%) males and 658 (69.3%) female citizens from various areas of Lebanon. The participants were distributed among various age groups, where 246 (25.89%) aged between 18 and 25 years, 286 (30.10%) aged between 30 and 35 years of age, and 166 (17.47%) aged over 40 years of age. The descriptive statistics showed that 509 (53.6%) of the participants did not work in the health care sector, while 441 (46.4%) were health care workers. The respondents were asked about their quarantine patterns and practices, such as potential exposure sources. The results showed that 441 (46.4%) of the participants reported that the fact of being a health care worker in the time of the spread of COVID-19 is in itself a major source of exposure, while 334 (35.2%) citizens reported that they are practicing home quarantine and that the only sources they might contract COVID-19 from would be their household. In addition, 106 (11.2%) of the participants reported that they are still going to work and that the source of exposure might be a coworker, while 46 (4.8%) indicated that being a patient procuring health care services at a hospital is a major source of exposure. In all, 908 (95.6%) of the respondents reported that they have not been in contact with any suspected COVID-19 case 4 weeks ago, 558 (59.7%) of them reported that leave home during the quarantine period and they are not strictly abiding by home quarantine, where 338 (35.6%) of them left home to go to the supermarket while 258 (27.2%) broke their quarantine to go to work. The average duration for leaving home quarantine was 2.88 hours per day (Table 1).

**Table 1.** Sociodemographic data and quarantine practices.

	*n*	%
Age		
18–25 years old	246	25.89
25–30 years old	110	11.6
30–35 years old	286	30.10
35–40 years old	142	14.94
>40 years old	166	17.47
Gender		
Male	292	30.7
Female	658	69.3
Occupation		
Non–health care worker	509	53.6
Health care worker	441	46.4
Potential exposure sources		
Household	334	35.2
Patient	46	4.8
Health care facility visitor	22	2.3
Coworker	106	11.2
Travel	1	0.11
Health care provider	441	46.4
Have you been in contact with any suspected COVID-19 case 4 weeks ago?		
No	908	95.6
Yes	42	4.4
Do you leave home during the quarantine period?		
No	392	41.3
Yes	558	58.7
Reason for leaving home during the quarantine		
Work	258	27.2
Supermarket	338	35.6
Pharmacy	22	2.3
None	332	34.9

### PTSD symptoms

The PCL-C was used in order to measure the prevalence of PTSD symptoms among the citizens during the times of COVID-19 quarantine in Lebanon on two occasions; 2 weeks after the start of quarantine and then 4 weeks after that point of time. Any response that ranged between 1 and 2 on the Likert-type scale was considered asymptomatic, while the responses that ranged between 3 and 5 were considered symptomatic. The majority of the respondents were not symptomatic upon answering the PCL-C.

During the second week of quarantine, the highest reported symptom among the B items was ‘Feeling very upset when something reminded you of a stressful experience from the past’ where 316 (33.2%) were symptomatic, followed by ‘Repeated, disturbing memories, thoughts or images of a stressful experience from the past’ where 204 (21.47%) were symptomatic. On the level of C items, the most reported statement was ‘Feeling distant or cut off from other people’ were 412 (43.36%) were symptomatic, followed by ‘Feeling as if your future will somehow be cut short’ were 352 (37.05%) were symptomatic. As for the D items, the most reported symptom was Being ‘super alert’ or ‘watchful on guard’ where 366 (28.52%) were symptomatic, followed by ‘Feeling irritable or having angry outbursts’ reported by 352 (37.05%) of the respondents (Table 2).

**Table 2.** Post-traumatic Stress Disorder Checklist (Week 2).

	Response	Asymptomatic				Symptomatic					
		Not at all (1)		A little bit (2)		Moderately (3)		Quite a bit (4)		Extremely (5)	
		*n*	%	*n*	%	*n*	%	*n*	%	*n*	%
B	Repeated, disturbing memories, thoughts, or images of a stressful experience from the past?	390	41.1	356	37.5	136	14.3	68	7.2	0	0
B	Repeated, disturbing dreams of a stressful experience from the past?	514	54.1	264	27.8	104	10.9	42	4.4	26	2.7
B	Suddenly acting or feeling as if a stressful experience were happening again (as if you were reliving it)?	470	49.5	270	28.4	134	14.1	56	5.9	20	2.1
B	Feeling very upset when something reminded you of a stressful experience from the past?	298	31.4	336	35.4	170	17.9	76	8.0	70	7.4
B	Having physical reactions (e.g. heart pounding, trouble breathing or sweating) when something reminded you of a stressful experience from the past?	500	52.6	256	26.9	92	9.7	52	5.5	50	5.3
C	Avoid thinking about or talking about a stressful experience from the past or avoid having feelings related to it?	370	38.9	262	27.6	162	17.1	80	8.4	76	8.0
C	Avoid activities or situations because they remind you of a stressful experience from the past?	558	58.7	180	18.9	122	12.8	50	5.3	40	4.2
C	Trouble remembering important parts of a stressful experience from the past?	572	60.2	210	22.1	102	10.7	40	4.2	26	2.7
C	Loss of interest in things that you used to enjoy?	388	40.8	262	27.6	154	16.2	88	9.3	58	6.1
C	Feeling distant or cut off from other people?	288	30.3	250	26.3	172	18.1	140	14.7	100	10.5
C	Feeling emotionally numb or being unable to have loving feelings for those close to you?	448	47.2	206	21.7	128	13.5	88	9.3	80	8.4
C	Feeling as if your future will somehow be cut short?	298	31.4	300	31.6	146	15.4	124	13.1	82	8.6
D	Trouble falling or staying asleep?	422	44.4	232	24.4	122	12.8	70	7.4	104	10.9
D	Feeling irritable or having angry outbursts?	322	33.9	276	29.1	168	17.7	98	10.3	86	9.1
D	Having difficulty concentrating?	362	38.1	282	29.7	156	16.4	76	8.0	74	7.8
D	Being ‘super alert’ or watchful on guard?	326	34.3	258	27.2	208	21.9	84	8.8	74	7.8
D	Feeling jumpy or easily startled?	400	42.1	308	32.4	150	15.8	56	5.9	36	3.8

During the fourth week of quarantine, the symptoms of PTSD appear to be more prevalent in comparison to the numbers reported during the second week of quarantine, where on the level of the B item ‘Feeling very upset when something reminded you of a stressful experience from the past’, 595 (62.63%) were symptomatic higher than 316 (33.2%) reported during the second week. On the level of the C item ‘Feeling distant or cut off from other people’, 655 (68.94%) were symptomatic higher than 412 (43.36%) during the second week. Further on the level of the D item, ‘Being super alert or watchful on guard’, 672 (66%) were symptomatic, higher than 366 (28.52%) during the second week. The details are provided in Table 3.

**Table 3.** Post-traumatic Stress Disorder Checklist (Week 4).

	Response	Asymptomatic				Symptomatic					
		Not at all (1)		A little bit (2)		Moderately (3)		Quite a bit (4)		Extremely (5)	
		*n*	%	*n*	%	*n*	%	*n*	%	*n*	%
B	Repeated, disturbing memories, thoughts, or images of a stressful experience from the past?	242	25.47	416	43.79	113	11.89	122	12.84	57	6.00
B	Repeated, disturbing dreams of a stressful experience from the past?	318	33.47	240	25.26	235	24.74	84	8.84	73	7.68
B	Suddenly acting or feeling as if a stressful experience were happening again (as if you were reliving it)?	291	30.63	278	29.26	235	24.74	97	10.21	49	5.16
B	Feeling very upset when something reminded you of a stressful experience from the past?	130	13.68	225	23.68	274	28.84	198	20.84	123	12.95
B	Having physical reactions (e.g. heart pounding, trouble breathing or sweating) when something reminded you of a stressful experience from the past?	376	39.58	173	18.21	184	19.37	113	11.89	104	10.95
C	Avoid thinking about or talking about a stressful experience from the past or avoid having feelings related to it?	221	23.26	136	14.32	265	27.89	196	20.63	132	13.89
C	Avoid activities or situations because they remind you of a stressful experience from the past?	447	47.05	102	10.74	177	18.63	127	13.37	97	10.21
C	Trouble remembering important parts of a stressful experience from the past?	352	37.05	107	11.26	245	25.79	134	14.11	112	11.79
C	Loss of interest in things that you used to enjoy?	131	13.79	95	10.00	281	29.58	256	26.95	187	19.68
C	Feeling distant or cut off from other people?	163	17.16	132	13.89	279	29.37	235	24.74	141	14.84
C	Feeling emotionally numb or being unable to have loving feelings for those close to you?	236	24.84	187	19.68	207	21.79	199	20.95	121	12.74
C	Feeling as if your future will somehow be cut short?	193	20.32	182	19.16	256	26.95	187	19.68	132	13.89
D	Trouble falling or staying asleep?	287	30.21	165	17.37	197	20.74	113	11.89	188	19.79
D	Feeling irritable or having angry outbursts?	276	29.05	165	17.37	195	20.53	136	14.32	178	18.74
D	Having difficulty concentrating?	287	30.21	132	13.89	248	26.11	152	16.00	131	13.79
D	Being ‘super alert’ or watchful on guard?	214	22.53	109	11.47	329	34.63	132	13.89	166	17.47
D	Feeling jumpy or easily startled?	221	23.26	163	17.16	259	27.26	185	19.47	122	12.84

Independent *t*-tests and analysis of variance (ANOVA) were carried out to determine if there’s a difference in the prevalence of PTSD symptoms among the civilians according to various characteristics and quarantine patterns. The results showed that there was no difference among genders (*p* = .07), and among occupations, whether the respondent was a health care worker or not (*p* = .34). Age (*p* = .15) and leaving home during quarantine or not (*p* = .77) did not result in a significant difference in PTSD symptoms; however, the possible sources of exposure to COVID-19 did make a difference (*p* = .02) (Table 4).

**Table 4.** The difference in PTSD symptoms according to respondent characteristics and quarantine patterns.

	*t*	Mean	*SD*	*p*-value
Gender				
Male	−1.37	0.96	0.73	.07
Female	−1.35	1.03	0.71	
Leaving home during the quarantine				
Yes	−1.56	0.97	0.03	.77
No	−1.56	1.04	0.03	
Contact with COVID-19 cases				
Yes	−0.78	1.01	0.02	.59
No	−0.80	1.10	0.10	
Occupation				
Health care	−1.57	0.98	0.03	.34
Non–health care	−1.57	1.05	0.03	
	*df*	*M*	*F*	*p*-value
Age				
Between groups	4	0.87	1.68	1.15
Within groups	945	0.51		
Potential exposure sources				
Between groups	5	1.36	2.64	**.02**
Within groups	944	0.51		

### Predicting factors of PTSD

Furthermore, regression analysis was carried out and neither gender (*p* = .13), age (*p* = .19), occupation (*p* = .96), nor potential sources of exposure (*p* = .48) and quarantine practices such as leaving home or not (*p* = .16) have been predictors of PTSD symptoms (Table 5).

**Table 5.** Predictors of Post-traumatic Stress Disorder symptoms.

	*B*	Std. error	β	*t*	*p*-value
Age	0.07	0.05	0.04	1.49	.13
Gender	0.02	0.02	0.04	1.30	.19
Occupation	0.00	0.11	0.00	0.04	.96
Potential exposure sources	0.01	0.02	0.05	0.70	.48
Contact with COVID-19 cases	0.07	0.11	0.02	0.64	.51
Leaving home during the quarantine	0.08	0.05	0.05	1.39	.16

## Discussion

For the past month in the least, the universal community has closed borders with each other in an attempt to control the outbreak of the COVID-19 pandemic. Quarantine and social distancing measures have been presented as an obligatory option that is being enforced by armed forces in Lebanon in order to avoid the flooding of the health care system.

The results of this study showed that a noteworthy proportion of the participants have reported PTSD symptoms during quarantine, where the most reported symptoms were ‘feeling distant or cut off from other people’, ‘feeling very upset when something reminded you of a stressful experience from the past’, and ‘repeated, disturbing memories, thoughts or images of a stressful experience from the past’. This proves a noteworthy psychological impact of quarantine among a considerable proportion of the Lebanese community. The number of symptomatic citizens increased substantially during the fourth week of quarantine, which proves a higher psychological influence of quarantine. This is consistent with a previous study where a proportion of Australian horse breeders quarantined for many weeks owing to an epidemic of equine flu recorded elevated psychological trauma during the epidemic. The symptomatic proportion relative to the Australian population was similar to that proportion relevant to the Lebanese citizens in our study (Taylor et al., 2008). Our study did not show a significant difference between health care workers and non–health care workers regarding PTSD symptoms even though a higher mean of symptoms were recorded by health care providers and this can be explained by the fact that the quarantine is still in its second week, the sample is rather small, and that the health care system and the medical and nursing staff in Lebanon are receiving governmental and non-governmental support to fight COVID-19, in addition to the flexibility of those health care workers in leaving the hospitals to go home and are not on strict isolation or quarantine. A similar study was carried out among hospital staff workers who reported high depressive symptoms due to quarantine (Lee et al., 2018).

However, inconsistent with our findings, a previous study has shown that health care providers showed more severe symptoms of PTSD upon quarantine than the general public (Huremović, 2019). However, another study did agree with our findings and has shown that being a health care worker in quarantine was not associated with higher psychological symptoms (Hawryluck et al., 2004). Thus supporting our results, a previous study (Hossain et al., 2020) also found similar results to our research, where it evaluated PTSD prevalence among children and parents who have been quarantined, and it revealed that a notable percentage of those who have abided by quarantine have shown symptoms that pertain of a PTSD diagnosis. Other descriptive studies addressing psychological distress among the quarantined population have shown a high rate of low mood and irritability (Lee et al., 2018) in addition to emotional disturbance, depression, and stress (Yoon et al., 2016). A similar condition was reported during SARS outbreak, where civilians had to practice certain measures of quarantine and social distancing. Several reports have shown close numbers to our results, where studies on quarantined people who have experienced close encounters to potential carries of the SARS have indicated the prevalence of psychological distress such as fear, nervousness, sadness, and guilt (Brooks et al., 2020).

Not only quantitative studies have reported similar results to ours, but rather qualitative research have also indicated that psychological distress can result from quarantine practices such as confusion, fear, anger, grief, numbness and anxiety-induced insomnia (Caleo et al., 2018; Desclaux et al., 2017; Pellecchia et al., 2015). In a previous research paper that has examined psychological responses during and after quarantine has shown comparable numbers of individuals reporting anxiety symptoms and feelings of anger, whereas 4–6 months after quarantine, these symptoms had diminished (Jeong et al., 2016). Our results found that the respondents reported being alert and on guard all the time, and this was consistent with a previous study which found that due to SARS quarantine, a notable proportion of people tried to not get in contact with people coughing or sneezing, avoided crowds and stayed home even weeks after the quarantine was over, some even kept on obsessive hand hygiene (Reynolds et al., 2008). The finding of this study examined certain characteristics such as age, gender, and quarantine practices to be predictive of PTSD symptoms, yet the result was negative. This was inconsistent with previous research (North et al., 2012) that has found that younger age and lower educational attainment were predictive of PTSD due to quarantine, while another study (Hawryluck et al., 2004) found that age was not associated with psychological distress, and this is consistent with our findings which can be explained that the majority of our sample were aged between 18 and 25 years.

### Limitations

This study has been conducted among a proportionately small sample of the general Lebanese public, during the first 2 weeks of quarantine in Lebanon, a period during which the public was relatively still going out from the homes, and before the armed forces started enforcing strict laws and punishments to people breaking the regulations of the partial embargo. These conditions might have limited the prevalence of various results as well as might limit the generalization, even though the sample was distributed among the various provinces.

## Conclusion

Upon the global outbreak of COVID-19, quarantine has become a necessity for survival and avoiding the coronavirus death toll to climb as a result of flooding the health care system, especially in Lebanon where until recently only 400 mechanical were functional thus limiting the number of critical COVID-19 cases to be accepted for treatment. On the contrary, Quarantine practices among the general public have started to give rise to psychological distress and specifically PTSD symptomatology among a notable proportion of the public which is suspected to augment in the coming days, especially now that the Lebanese people are abiding more and more by the home quarantine and social distancing upon the strict measures of the government.

### Recommendations

The authors recommend repeating the study after the end of quarantine thus to compare the psychological distress during and the persistence of such symptoms after this phase, among a larger representative sample, recruiting especially various health care providers to evaluate their psychological affection due to the application of quarantine that has been further decided by the Lebanese ministry of health.
